# Fibrinogen-Like Protein 2/Fibroleukin Induces Long-Term Allograft Survival in a Rat Model through Regulatory B Cells

**DOI:** 10.1371/journal.pone.0119686

**Published:** 2015-03-12

**Authors:** Séverine Bézie, Elodie Picarda, Laurent Tesson, Karine Renaudin, Justine Durand, Séverine Ménoret, Emmanuel Mérieau, Elise Chiffoleau, Carole Guillonneau, Lise Caron, Ignacio Anegon

**Affiliations:** 1 INSERM UMR 1064-ITUN, Centre Hospitalier Universitaire de Nantes, Faculté de Médecine, Nantes, France; 2 Centre Hospitalier Universitaire de Nantes, Faculté de Médecine. Nantes, France; Université Paris Descartes, FRANCE

## Abstract

We previously described that in a rat model of heart transplantation tolerance was dependent on CD8^+^CD45RC^low^ Tregs that over-expressed fibrinogen-like protein 2 (FGL2)/fibroleukin. Little is known on the immunoregulatory properties of FGL2. Here we analyzed the transplantation tolerance mechanisms that are present in Lewis 1A rats treated with FGL2. Over-expression of FGL2 *in vivo* through adenovirus associated virus -mediated gene transfer without any further treatment resulted in inhibition of cardiac allograft rejection. Adoptive cell transfer of splenocytes from FGL2-treated rats with long-term graft survival (> 80 days) in animals that were transplanted with cardiac allografts inhibited acute and chronic organ rejection in a donor-specific and transferable tolerance manner, since iterative adoptive transfer up to a sixth consecutive recipient resulted in transplantation tolerance. Adoptive cell transfer also efficiently inhibited anti-donor antibody production. Analysis of all possible cell populations among splenocytes revealed that B lymphocytes were sufficient for this adoptive cell tolerance. These B cells were also capable of inhibiting the proliferation of CD4^+^ T cells in response to allogeneic stimuli. Moreover, gene transfer of FGL2 in B cell deficient rats did not prolong graft survival. Thus, this is the first description of FGL2 resulting in long-term allograft survival. Furthermore, allograft tolerance was transferable and B cells were the main cells responsible for this effect.

## Introduction

In a model of cardiac allotransplantation in rats, we have previously shown that blocking CD40-CD40L binding by treatment with recombinant CD40Ig induces long-term graft survival [1]. CD40Ig is composed by the extracellular portion of murin CD40 fused to the Fc portion of an human immunoglobulin [1]. CD40Ig is produced *in vivo* following gene transfer with an adenoviral vector encoding for CD40Ig (AdCD40Ig) into the cardiac graft [1,2]. In this transplantation tolerance model, alloantigen-specific CD8^+^CD45RC^low^ regulatory T cells (CD8^+^ Tregs) were generated [2,3]. Rat CD8^+^ Tregs were capable of transferring transplantation tolerance through IFNγ production, which induces IDO in different cell types [2]. Nevertheless, other mechanisms are also active and in general little is known about the mechanisms and molecules involved in the function of these or other CD8^+^ Tregs [4].

Transcriptomic comparison of CD8^+^ Tregs from rats treated with CD40Ig vs. naive rats, revealed fibrinogen-like protein 2/fibroleukin (FGL2) over-expression [5]. Some studies have shown that cell membrane-associated FGL2 has a prothrombinase activity involved in acute graft rejection when expressed by the graft [6,7]. In contrast, several studies have also described that soluble FGL2 has immunosuppressive functions, such as that mediated by mouse and human CD4^+^CD25^+^Foxp3^+^ Tregs [8–11]. Furthermore, FGL2 protein administration prolonged skin allograft survival without tolerance induction [12]. In the rat, we have shown that FGL2 is involved in CD8^+^ Treg suppression of *in vitro* CD4^+^ T cell responses [5]. Nevertheless, the contribution of FGL2 by itself in this rat cardiac allograft survival *in vivo* and in a more general way in allotransplantation remains to be determined.

Different types of immune cells have immunosuppressive and tolerogenic properties, the most studies until now have been on Treg cells and tolerogenic dendritic cells (DCs) and in recent years also regulatory B cells (Bregs) have received attention [13–15]. Understanding the generation and function of these tolerogenic cells could help to find new tolerance strategies to be applied in pathophysiological situations in which immune responses need to be inhibited in the most antigen-specific way as possible.

In this study we show that *in vivo* FGL2 overexpression through gene transfer results in inhibition of allograft rejection and generation of Bregs.

## Materials and Methods

### Ethics statement

This study was carried out in strict accordance with the protocol approved by the Committee on the Ethics of Animal Experiments of Pays de la Loire (permit number CEEA.2011.44). Lewis 1A, Lewis 1W, and Brown Norway rats were purchased from Janvier Labs (Saint-Berthevin, France). All surgery was performed under O2-isoflurane-N2O anesthesia, and analgesics were used post-operatively to minimize pain.

### Cardiac transplantation models

Heterotopic heart allotransplantation was performed between MHC complete incompatible male LEW.1W (donors, haplotype RT1u) and LEW.1A (recipients, haplotype RT1a) 8 to 12-week-old rats as previously described [2]. Third-party grafts were from the Brown-Norway strain (haplotype RT1n). Rats deficient for B cells due to a knockout of the *Igm* gene using zinc-finger nucleases have been already described [16]. These animals lack differentiated B cells and are unable to generate antibodies even after allogeneic immunization [16].

Equivalent dose of AAVFGL2 or AAVnull (see below) viral genome (3x10^12^vector genomes/kg) was administered *i*.*v*. (in a volume of 500 μl) parallelly in experimental and control groups of 2 or 3 4-week-old LEW.1A WT or Igm KO rats one month before the transplantation. Cardiac graft survival was evaluated by palpation through the abdominal wall.

### AAV generation

The rat FGL2 plasmid was constructed by positioning the respective complete cDNA sequences downstream of a RSV promotor, the Igκ signal peptide and a Flag sequence **(S1A Fig.)**. The control GFP vector and non coding vectors contained the RSV promotor and the control non-coding vector contained no cDNA. The FGL2 and GFP plasmids were first tested in HEK293T cells transfected with lipofectamine reagent (Life Technologies, Carlsbad, CA). GFP expression was observed 24h later. Cells and supernatants were analyzed for FGL2 expression by FACS and western blot 48h after transfection. The plasmids were then used to produce AAV vectors of serotype 2/8 (LTG platform, INSERM UMR 1089, Nantes), respectively AAV-FGL2, AAV-GFP and AAVnull. Titration of AAV was determined by slot blot detection of viral genomes (vg/ml). HEK293T cells were transduced with 10^2^ to 10^4^ vector genome copies/cell of AAV-FGL2 or AAV-GFP and 5x10^3^ for AdLacZ. Twenty-four hours later, since GFP was expressed, cells were harvested and analyzed for FGL2 expression by PCR and by FACS before adenoviral-induced cell apoptosis. AAV vectors were administered in rats as described above. Liver, spleen and graft samples were taken for FLAG-FGL2 transcript expression 30, 75, and 120 days after infection and a blood sample was taken for donor-specific alloantibody quantification at day 120 post transplantation.

### Quantitative RT-PCR

The isolation and retrotranscription of mRNA as well as the quantification of specific mRNA levels using SYBR green technology after normalization to HPRT values have been described [2]. The sequence of primer pairs for rat *Fgl2* RT-PCR were 5’-CAAGAACACAACCAGCCAAATCC-3’ (forward) and 5’-CCCAGCCAAAATTCTCGTTCAA-3’ (reverse) (**S1E Fig.**). The sequence of primer pairs for FLAG-FGL2 PCR were 5’-GATTACAAGGACGACGATGACAAG-3’ (forward) and 5’-CCCAGCCAAAATTCTCGTTCAA-3’ (reverse) for first PCR, and 5’-TGACTGAAGGGCTGGAGGA-3’ (forward) and 5’-GTCCGCACTTCTTTGAGCAC-3’ (reverse) for nested PCR (**S1G Fig.**). PCR products were analyzed by electrophoresis on agarose gel or by LabChip DX (Caliper Life Sciences, Hopkinton, Massachusetts).

### Generation of a polyclonal rat FGL2 antibody

Rabbits were immunized against two rat FGL2-specific peptidic sequences CKLQADEHPDPGGN and CYKFSFKKAKMMIRPKSFKP (PolyPeptide Laboratories, France). Low homology was only found with rat ASH2, and LZTS2 genes (66 and 63%, respectively). Rat FGL2 antibodies were purified from serum with a peptide-coupled NHS-HiTrap column. Albumin contamination was removed with protein A columns. Eluates purity was checked by silver-nitrate coloration. Anti-FGL2 reactivity was tested against both rat FGL2 peptides used for Ab production by ELISA, against the human-recombinant FGL2 protein (Abnova, Taïwan) by western blot and against the rat FGL2 protein expressed in cells transfected by a plasmid encoding FGL2 (plFGL2 see above) by FACS.

### Monoclonal antibodies and flow cytometry

The mouse antibodies used for the detection of rat T cells (R7/3), CD4^+^ cells (OX35), CD8^+^ cells (OX8), CD8^+^CD45RC^low^ cells (OX8, OX22), pDCs (85C7) [17] and CD45RA^+^ cells (OX33) were obtained, with the exception of 85C7, from the European Collection of Cell Culture (Salisbury, UK) and mAb were purified from supernatants followed by coupling to fluorochrome (Invitrogen, Cergy Pontoise, France). All biotin-labeled mAbs were visualized using Strepavidin-PE-Cy7 or Streptavidin-Alexa405 (BD Biosciences). FGL-2 was detected using a polyclonal antibody produced in house (see above for details) or mouse anti-human FGL2 cross-reacting with rat clone M02 (Abnova, Taiwan). A Canto II cytometer (BD Biosciences, Mountain View, CA) was used to measure fluorescence and data were analyzed using FLOWJO software (Tree Star, Inc. USA). Cells were first gated by their morphology and dead cells excluded by selecting DAPI negative viable cells.

### Mixed lymphocyte reaction

Naive Lewis 1A CD4^+^CD25^−^ T cells, Lewis 1W cDCs and pDC were sorted by FACS Aria as previously described [2,5] (**gating strategies in S2A Fig.**). 5x10^4^ CD4^+^CD25^−^ and 12500 pDC were cultured per well [5]. Human recombinant FGL2-GST protein was added to CD4^+^ T cells and pDC from 0.4 to 10μg/ml for analysis of suppressive activity. The suppressive activity of B cells was tested by adding CD45RA^+^ FACS-sorted B cells at a 1:1 ratio with CD4^+^CD25^−^ T cells. Relative proportion of dividing CD4^+^CD25^−^ T cells was analyzed by flow cytometry 6 days later, by gating on TCR^+^CD4^+^ cells (R7/3-APC, OX35-PECY7) among live cells and analysing CFSE labelling dilution (**S2B Fig.**).

### Adoptive cell transfer

Spleens were digested by collagenase D for 15 minutes at 37°C, stopped with 400 μl of 0.1mM EDTA, and red blood cells were lysed. Splenocytes were sorted as previously described [2,5] **(and as outlined in S3D Fig., purity > 99%)** by FACS Aria (BD Biosciences, Mountain View, CA) by gating on TCRαβ-APC (R7/3) and TCRγδ-FITC (V65), CD8^+^CD45RC^low^ T cells (OX8, OX22), pDC (85C7), or CD45RA-biotin-Strepavidin-PE-Cy7 (OX33) positive cells. Recipients that received splenocytes from FGL2-treated rats are defined as 1^st^ transferred and then iterative transfers were defined as 2^nd^-to 6^th^ transferred. Since, the phenotype or tolerogenic potential of splenocytes were not different among different iteratively transferred animals, we purified subtypes from all of them. Total splenocytes (1x10^8^ cells) and FACS Aria-sorted CD45RA^+^ B cells or T cells (equivalent numbers of B or T cells obtained each from 1x10^8^ splenocytes from the same donor, from 5–40 x10^6^ cells, 1^st^ or 3^rd^ transferred), CD8^+^CD45RC^low^ (5x10^6^ cells, 2^nd^ and 4^th^ transferred), or pDC (1x10^6^ cells, 2^nd^, 3^rd^ and 4^th^ transferred) were adoptively transferred *i*.*v*. the day before heart transplantation into naive LEW-1A recipients that had received 4.5 Gy of whole-body irradiation the same day.

### Donor specific alloantibody quantification

Donor spleens were digested by collagenase D, stopped with 400μl of 0.1mM EDTA, and red cells were lysed. Serum complement was inactivated by heating prior to incubation with donor splenocytes at a 1/8 dilution and revelation with either anti-rat IgG1 (MCA 194, Serotec), anti-rat IgG2a (MCA 278, Serotec), or anti-rat IgG2b (MCA195, Serotec) and anti-Ms Ig-488 as an isotype control (ref 11029, Serotec). A FACS Canto (BD Biosciences, Mountain View, CA) was used to measure fluorescence and data were analyzed using FLOWJO software (Tree Star, Inc. USA). Geometric mean fluorescence intensity (MFI) of tested sera was divided by the mean MFI values of 5 naive Lewis-1A sera used as controls.

### Statistical analysis

GraphPad Prism 4.0 (La Jolla, CA, USA) was used for statistical analysis. The Wilcoxon tests were used for analysis of coculture experiments, the Two-Way ANOVA test and Bonferroni post tests were used for donor-specific antibody analysis and splenocyte phenotype characterization, and the Log Rank (Mantel Cox) test was used to analyze survival rates. Statistical significance was defined as p<0.05.

## Results

### FGL2 induces long-term cardiac graft survival

To test whether FGL2 could induce long-term cardiac allograft survival, we expressed rat FGL2 *in vivo* using an AAV recombinant vector encoding for the cDNA of rat FGL2 tagged with a TAG epitope (AAV-FGL2). We confirmed the expression of the FGL2- plasmid (plFGL2), used afterwards to generate AAV-FGL2, in cell supernatants from transfected HEK293T cells by western blot using an in house generated rabbit polyclonal anti-rat FGL2 antibody and by FACS **(S1B, C, and D Fig.)**. FGL2 expression following AAV-FGL2 HEK293T cell transduction *in vitro* was confirmed by quantitative RT-PCR and FACS analysis **(S1E and F Fig.)**.

**Fig 1:**
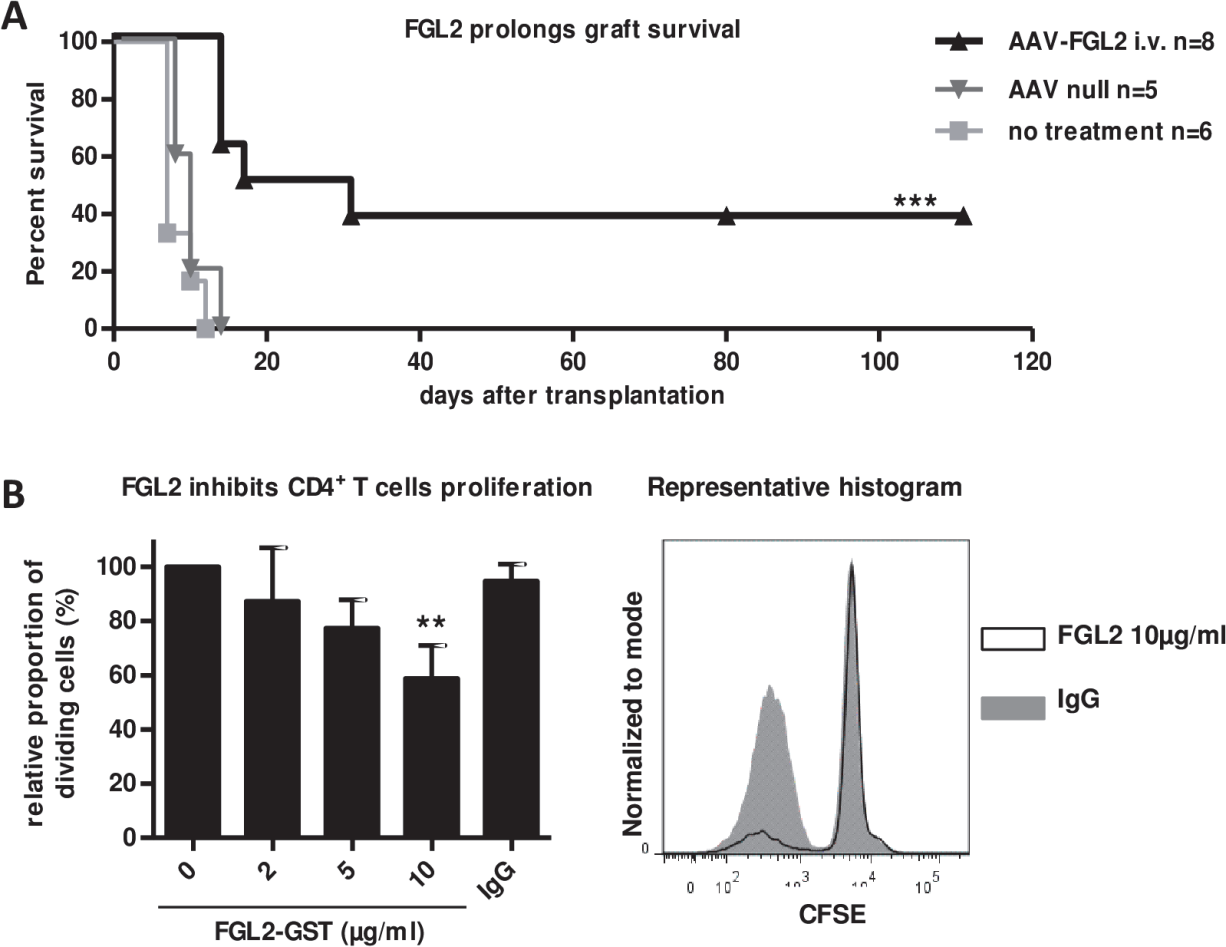
Over-expression of FGL2 *in vivo* prolongs cardiac allograft survival. **(A)** Cardiac graft recipients received intravenously 3x10^12^vector genomes/kg of AAV-FGL2 (▲ n = 8), or non coding AAV (▼ n = 5, 2 different experiments), and received a heterotopic transplant 30 days later. Graft survival was evaluated by palpation through the abdominal wall. Log-rank (Mantel-Cox) test ***p<0.*001* for AAV-FGL2 vs. AAV null controls. **(B)** Left: Relative proportion of dividing CD4^+^CD25^−^ T cells at day 6 in the presence of different concentrations of recombinant human FGL2-GST was evaluated by CFSE dilution by gating first on DAPI^-^ live cells and then on TCR^+^CD4^+^ cells **(S2B Fig.).** The negative control was purified rat IgG at 10 μg/ml (n = 4, ** *p*<0.01). Right: Representative histogram of relative proportion of dividing CD4^+^T cell in the presence of 10μg/ml FGL2-GST protein (black line) or IgG control (grey).

Allograft recipients received AAV-FGL2 by iv injection 30 days before the cardiac allograft, since we and others have shown maximal expression levels of sequences vectorized by AAV vectors around this time point [18,19]. We confirmed by PCR the presence of AAV-derived FLAG-FGL2 in liver and spleen at day 30 but not at day 75 after gene transfer in treated animals **(S1G Fig.).** Quantification of serum FGL2 was not possible despite considerable efforts since the commercially available monoclonal antibodies against human and rat FGL2 and the rabbit anti-rat FGL2 antibody we developed were not suitable for a sensitive FGL2 detection in serum (only detecting > 110 μg/ml). Intravenous injection of AAV-FGL2 resulted in a significant prolongation of graft survival, with 3/8 animals presenting long-term (>80 days) surviving grafts, vs. untreated (median survival time (MST) = 7) and non-coding AAV null-treated recipients (MST = 9) (**Fig. 1A**).

To confirm the suppressive functions of FGL2 in an MLR using rat cells, we co-cultured effector CD4^+^CD25^−^Tcells as responder cells, with allogeneic pDCs as stimulator cells, in the presence of a range of recombinant human FGL2 concentrations **(gating strategy of cell sorting in S2A Fig.)** since rat FGL2 is not available. Analysis of the proportion of dividing CD4^+^CD25^−^ T cells by CFSE labelling dilution showed a dose-dependent inhibition of proliferation by human FGL2 **(Fig. 1B)** at relatively high concentrations but since FGL2 was of human origin, affinity for rat FGL2 receptors may not be optimal.

### FGL2 induces splenic tolerogenic cells capable of transferable and dominant allogeneic graft acceptance

**Fig 2:**
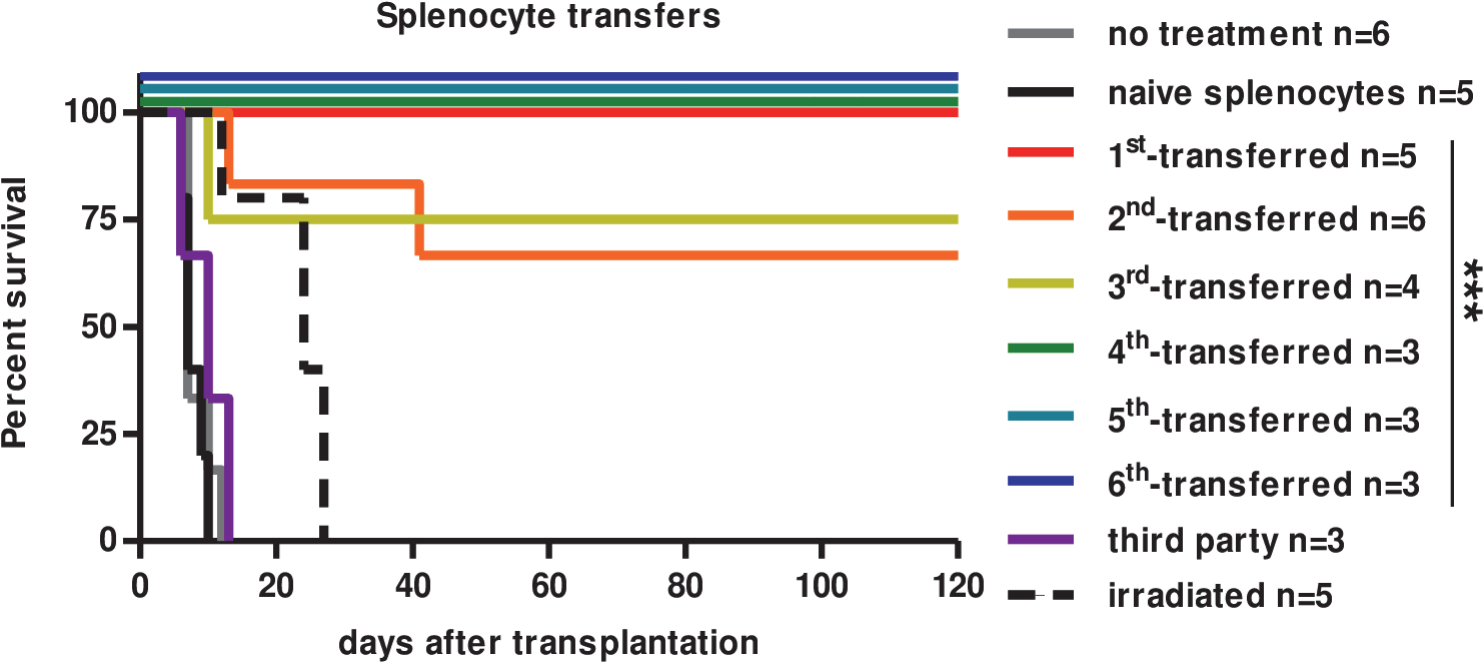
Splenocytes from AAVFGL2-treated rats with long-term surviving grafts transfer donor alloantigen-specific long-term graft survival in an iterative manner. Splenocytes from long-term AAV-FGL2-treated recipients were injected *i*.*v*. into sub-lethally irradiated recipients (LEW.1A) the day before heart allotransplantation (LEW.1W). Graft survival was evaluated by palpation through the abdominal wall. Total splenocytes (1x10^8^ cells) from long-term (≥120 days) AAV-FGL2-treated rats were adoptively transferred (1^st^-transferred, n = 5), and then total splenocytes (10^8^ cells) were iteratively transferred to 2^nd^- (n = 6), 3rd (n = 4), 4^th^ (n = 3), 5^th^ (n = 3) and 6^th^ (n = 3) LEW.1A recipients receiving LEW.1W hearts. Third-party grafts were from Brown-Norway origin and adoptive transfer of splenocytes from LEW.1W-transplanted animals did not inhibit acute rejection (third party, n = 3, performed in animals that received a second adoptive transfer). Splenocytes from naive non-transplanted rats did not inhibit acute rejection (naive splenocytes, n = 5) and non-irradiated non-transferred recipients (no treatment, n = 6) also showed acute rejection. Irradiation alone without cell transfer delays graft survival but does not prevent graft from rejection (irradiated, n = 5). All groups were compared to irradiated animals transferred with naive splenocytes by Log-rank (Mantel-Cox) Test (*p value* ***<0.001).

To determine whether FGL2 over-expression resulted in the generation of a tolerogenic cell population, splenocytes from AAV-FGL2-treated rats with long-term surviving allografts were transferred to sub-lethally irradiated recipients, the day before the transplant. It is important to note that serotype AAV2 *i*.*v*. injection transduces mostly hepatocytes and lung cells and in the spleen some macrophages but little or not lymphoid cells, as it is the case also for other AAV serotypes [18]. Adoptive cell transfer of splenocytes resulted in long-term graft survival for all (5/5) recipient animals, contrary to untreated grafted rats (0/6) (MST = 7) and rats transferred with splenocytes from naive rats (0/5) (MST = 7) (**Fig. 2**). Moreover, splenocytes from these adoptively transferred tolerant recipients were again capable of transferring tolerance for at least six cycles of splenocyte transfer resulting in long-term graft survival between 66% (4/6 rats) and 100% (3/3) of grafted hearts animals (**Fig. 2**). Adoptive transfer of long-term allograft survival was donor-specific since 3/3 hearts of Brown-Norway third-party origin were rejected in secondary transferred recipients (MST = 10). Thus, splenocytes from FGL2-treated rats contained a cell population capable of inducing transferable and dominant tolerance in a donor-specific manner.

### Alloantibody levels analysis

**Fig 3:**
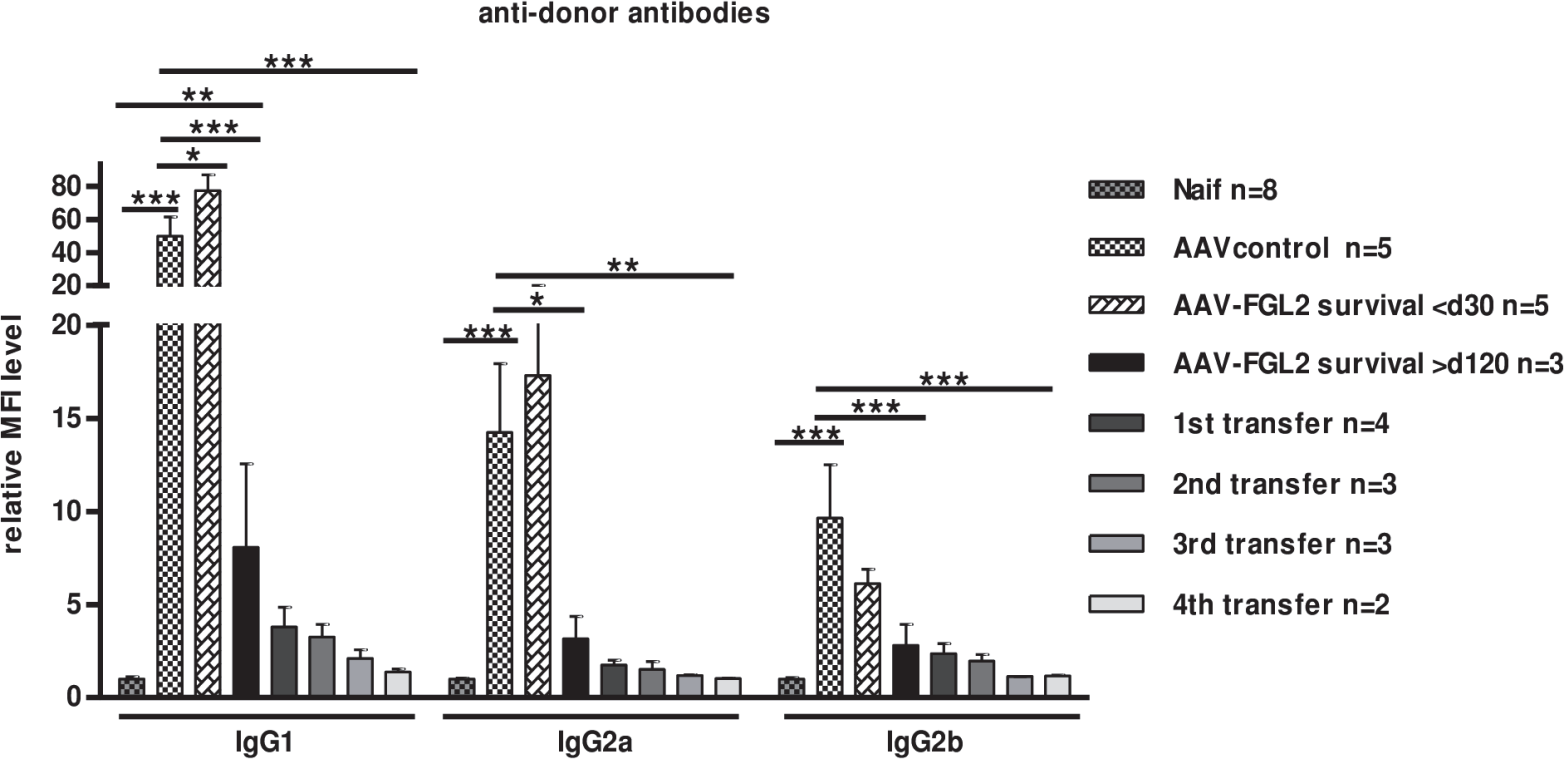
Alloantibody production was suppressed after AAV-FGL2 treatment and adoptive transfer of splenocytes. Sera were collected from naive rats, or at the moment of rejection from rats treated with AAV-control or AAV-FGL2 (rejecting at < 30 days or > 120 days after transplantation) or receiving adoptive transfers (> 120 days after transplantation). Levels of donor-specific IgG1, IgG2a, and IgG2b antibodies were evaluated by cytofluorimetry and normalized to serum from naive rats (MFI / MFI syngeneic). Two way Anova, Bonferroni post test *p value* * <0.5; ** <0.01; ***<0.001.

Donor-specific alloantibody levels of the IgG1, IgG2a and IgG2b isotypes were all drastically reduced in AAV-FGL2-treated recipients with long-term surviving grafts vs. AAV-null-treated rat controls and also vs. AAV-FGL2 treated rats that rejected their grafts early (<30 days) **(Fig. 3).** Recipients with adoptively transferred splenocytes showed a further decrease in alloantibody levels, including IgG1, IgG2a or IgG2b, resulting in levels significantly lower than those of AAV-FGL2-treated rats and comparable to naive animals **(Fig. 3)**. This indicates that FGL2 treatment and the tolerogenic cells generated by FGL2 globally inhibited anti-donor antibody immune responses which in the rat are controlled for the IgG2b isotype by CD4^+^ Th1 and for the IgG2a and IgG1 isotypes by Th2 cells [20,21].

### FGL2 induces regulatory B cells

In order to identify the cell populations involved in transferable and dominant tolerance, we first analyzed the phenotype of FGL2-treated and transferred splenocytes vs. splenocytes from naive animals. No statistically significant changes were seen among CD4^+^ T, CD8^+^ T, CD8^+^CD45RC^low^ T, CD8^+^CD45RC^high^ T, CD45RA^+^ B cells, CD4^+^CD25^+^Foxp3^+^ T cells or pDCs (**S3A and B Fig.**).

**Fig 4:**
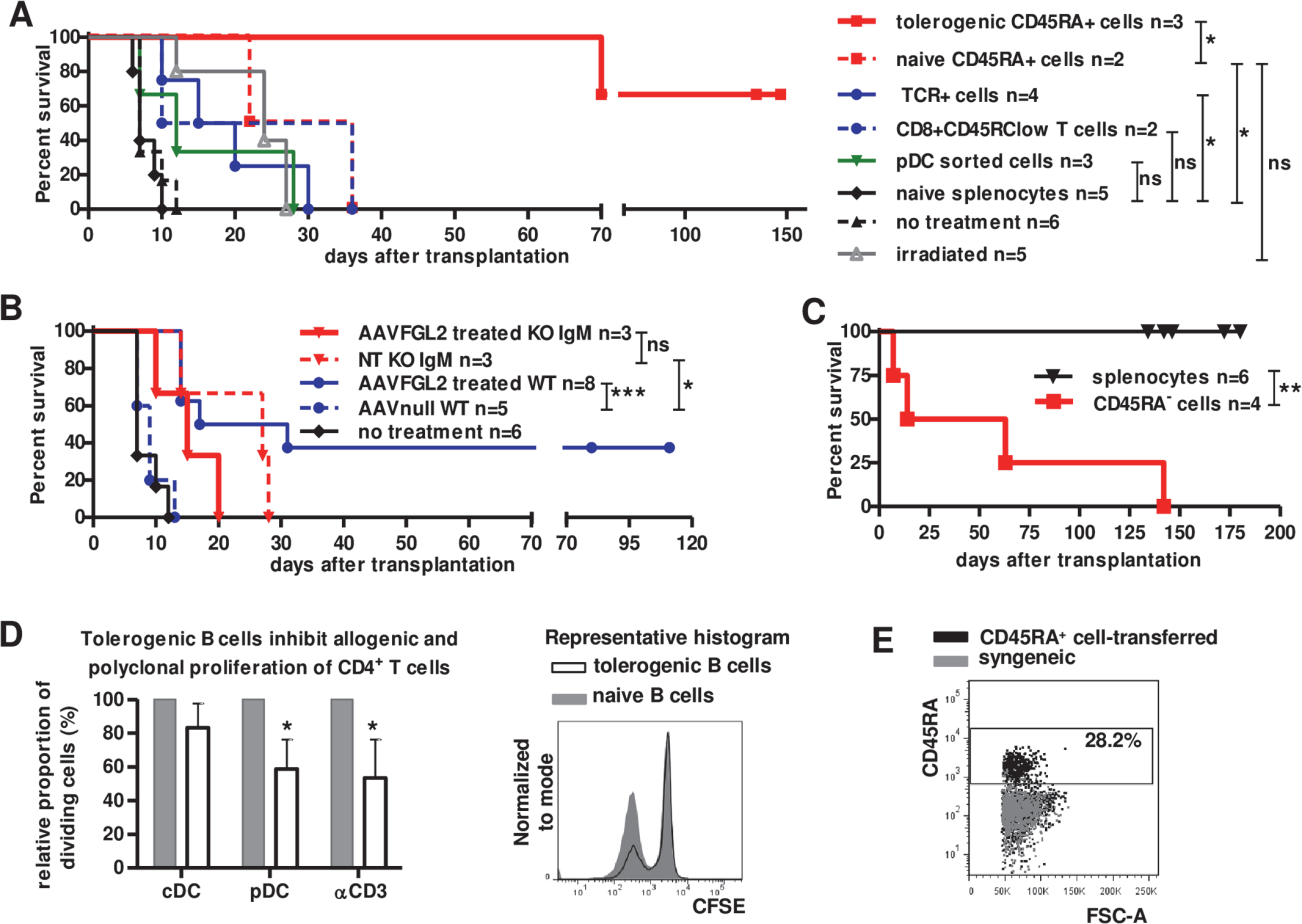
Adoptive transfer of B cells transfers tolerance. Cells were sorted by FACS Aria **(sorting strategy displayed in S3D and E Fig.)** from the spleen of tolerant rats (>100 days after the graft) that had received a transfer of splenocytes from a previously tolerant recipient and adoptively transferred to sub-lethally irradiated recipients the day before the transplant. **(A)** B cells (CD45RA^+^, n = 3), T cells (TCR^+^, n = 4), CD8^+^ Tregs (CD8^+^CD45RC^low^, n = 2), pDCs (mAb 85C7^+^ n = 3) Groups are compared with each other and to irradiated animals transferred with naive splenocytes (naive splenocytes, n = 5) by Log-rank (Mantel-Cox) Test *p* <0.05*; p<0.01**; p<0.001***. **(B)** Wild type (WT) and B cell-deficient *Igm* knockout (KO) rats were treated with AAV-FGL2 (n = 8 and 3, respectively), AAV-null (n = 5) or untreated (NT, n = 3), and analyzed for graft survival. **(C)** Splenocytes from adoptively-transferred tolerant rats were depleted in CD45RA^+^ B cells (CD45RA^−^ cells) or not (splenocytes) and transferred to new irradiated recipients. Log-rank (Mantel-Cox) Test p<0.01**. **(D) Left:** A fraction of the transferred tolerogenic CD45RA^+^ B cells was tested for inhibition of CFSE-labeled CD4^+^CD25^−^ T cell proliferation in response to allogeneic LEW.1W cDCs, pDCs (stimulator/effector ratio of 1:4) or anti-CD3 at day 6 of culture. Shaded grey: naive CD45RA^+^ B cells n = 3, black line: tolerogenic CD45RA^+^ B cells n = 4. **Right**: Representative histogram of one proliferation assay of CD4^+^CD25^−^ T cells with allogeneic pDCs and CD45RA^+^ B cells from naive (shaded grey) or splenocyte-transferred tolerant rats (black line). **(E)** Graft infiltrating cells were analyzed for the presence of CD45RA^+^ cells in graft of rats transferred with B cells, at days 100 after the graft, as compared with syngeneic grafts (n = 3).

We then purified different spleen cell populations and performed adoptive transfer experiments **(gating strategies in S3C and D Fig.)**. T cells significantly prolonged graft survival with a median of 17.5 days vs. 7 days for transfer of naive splenocytes, but this was not sufficient to prevent acute graft rejection. Similarly, neither CD8^+^CD45RC^low^ T cells nor pDC transfer were sufficient to induce long term graft survival (MST = 23 and 12 days respectively) (**Fig. 4A**). In view of these results, we analyzed the tolerogenic potential of B cells *in vivo*. CD45RA^+^ B cells from adoptively transferred rats with long-term surviving grafts were sorted by FACS Aria (**S3C and D Fig.**) and transferred to naive rats 12–24h before transplantation. Transfer of CD45RA+ B cells from tolerant recipients resulted in a long-term graft survival (**Fig. 4A**). In contrast, transfer of CD45RA^+^ B cells from naive non-transplanted rats had no effect was not significant vs. the survival in irradiated non-transferred recipients (MST = 29 vs. 24, respectively) (**Fig. 4A**). Importantly, AAV-FGL2-treatment of B-cell deficient *Igm* KO rats failed to prolong graft survival (MST = 24 vs. 15) **(Fig. 4B).** Furthermore, adoptive transfer of splenocytes depleted of CD45RA^+^ B cells from recipients with long-term surviving allografts following adoptive transfer of splenocytes failed to impose long-term allograft survival **(Fig. 4C).** In these recipients, allograft survival was nevertheless prolonged (MST = 38.5 days vs. long-term survival for total splenocytes, p<0.05), indicating that other regulatory cells were induced, as suggested by the moderate prolongation of allograft survival with transfer of total T cells. Altogether, B cells are necessary for the tolerogenic effect of FGL2 and B cells are needed to actively transfer tolerance. The suppressive properties of B cells from splenocyte-transferred tolerating rats were also tested *in vitro*. In MLR experiments, CD45RA^+^ B cells isolated from rats with long-term surviving grafts inhibited CD4^+^ T cell proliferation in response to allogeneic pDCs or anti-CD3-induced proliferation, compared to CD45RA^+^ B cells from naive rats **(Fig. 4D)**.

Following the transfer of tolerogenic CD45RA^+^cells, a large proportion (mean+SD 28.2% ±6.43) of cells observed within the grafts were CD45RA^+^ whereas CD45RA^+^ cells were barely detectable in syngeneic grafts **(Fig. 4E)**. Altogether, these results support FGL2 as being a tolerogenic molecule that favors the emergence of regulatory B cells which are enriched in the graft.

## Discussion

In this study we describe for the first time the capacity of FGL2 to induce long-term allograft survival through active cellular tolerogenic mechanisms involving B cells.

FGL2 has a dual prothrombinase and immunoregulatory activity, depending on its expression at the cell membrane or as a secretory molecule, respectively. FGL2 prothrombinase activity has been postulated to directly convert prothrombin to thrombin [22,23] and has been associated with fibrin deposition in some pathological processes [6,7,24–26]. FGL2 also exists as a secretory molecule with immunoregulatory activity [12,27,28]. In transplantation, administration of soluble FGL2 was shown to double skin allograft survival but did not result in long-term survival [12]. Recently, FGL2 together with Foxp3 and killer cell lectin-like receptor G1, were shown to be over-expressed in models of liver and cardiac transplantation tolerance [29]. Mice deficient in FGL2 develop autoimmune glomerulonephritis [28]. FGL2 has been shown to be expressed by mouse [8,9] and human CD4^+^CD25^+^Foxp3^+^ Tregs [11] as well as in CD8^+^αα suppressive intraepithelial T cells [30]. FGL2 was also found to inhibit LPS-induced maturation of mouse myeloid BMDCs [27] and to suppress T cell proliferation and cytokine production, mainly of Th1 and Th17 cells but not of Th2 cells [11]. Contrary to these results, FGL2-deficient mice rejected cardiac and skin allografts with identical timing to controls and also showed normal immune responses to microorganisms inducing a type 1 immune response [31].

Increased *Fgl2* mRNA expression during the first days following transplantation in the grafts of AdCD40Ig-treated rats correlates with graft infiltration by FGL2-overexpressing CD8^+^ Tregs [5]. Since not all animals treated with AAV-FGL2 developed long-term surviving grafts, it is likely that the gene transfer system used is not optimal and that other gene transfer vectors or direct protein administration may be necessary. It is also to note that since our model of allotransplantation involves complete major but not minor histocompatibility antigens future studies will include animal models with combined minor and major histocompatibility antigens incompatibility as well as with transplantation of other organs or tissues.

Bregs have been described in autoimmunity, cancer and transplantation [32]. In transplantation, Bregs have been described in a mouse model treated with anti-CD45RB alone [33] or in combination with anti-TIM-1 antibody [34] and in a rat model treated with an analogue of the immunosuppressive drug deoxyspergualin [35]. The possibility of Bregs has been suggested in spontaneously tolerant kidney transplant patients [36,37]. Although Bregs have been already shown to transfer transplant tolerance [34,35], we show here the capacity of Bregs to induce repeated transferable and dominant tolerance. This phenomenon is similar to infectious tolerance, usually through induction of tolerogenic DCs that will tolerize new naive T cells [38] and until now described for CD4^+^ [39] and CD8^+^ Tregs [2]. Nevertheless, despite being able to transfer tolerance for at least six adoptive transfers, the absence of congenic markers does not allow to exclude the expansion of B cells present in the first adoptive transfer and thus the definition of our model as infectious tolerance. Bregs from autoimmune models express markers of marginal zone and relatively immature transitional 2 precursors [32,40] whereas in *Salmonella* infection, have shown a mature phenotype [41]. Some transplantation models have described Bregs as mature cells that are blocked in their differentiation [35,42]. Due to the lack of antibodies against useful B cell maturation markers (like CD23, CD38, and CD138) in the rat, it is difficult to directly compare rat B cells with their human or mouse counterparts. In our model, Bregs accumulated in the graft. Only a few previous studies have detected Bregs at the site of inflammation, and include models such as lupus and autoimmune colitis in mice [43,44].

FcγRIIB is one of the receptors of FGL2 expressed by conventional DCs, albeit the existence of other receptors is not excluded [12,28]. FcγRIIB was reported as a marker of tolerance in patients with a Breg signature [36,37] and a recent manuscript described Bregs that have high expression of FcγRIIB [45]. We have observed expression of FcγRIIB at similar levels in both rat B cells from naive and FGL2-treated rats as well as in pDCs **(S4A and B Fig., respectively).** Since both pDCs and B cells express FcγRIIB, further experiments will be needed to define whether the emergence of Bregs by FGL2 was the consequence of an indirect effect on B cells through pDCs and/or a direct effect on B cells.

The role of BCR signaling in Breg generation has been suggested by some studies but has not yet been formally proven [32]. Loss of Breg function with conserved antibody production has been described in a mouse EAE model [46]. Analogously, Breg function in the anti-CD45RB transplantation tolerance model was independent of antibody production [33]. Our results showing donor-specific suppression suggest that BCR recognition of donor antigens could be involved. Donor-specific recognition could occur through binding of IgM, IgD or IgG expressed by CD45RA^+^ Bregs in these animals (data not shown). Since alloantibody production was inhibited, it is possible that signaling through the BCR was modified by FGL2- FcγRIIB interaction, with decreased alloantibody production and simultaneous production of immunosuppressive molecules.

The mechanisms by which mouse or human Bregs inhibit T cell responses include the generation of CD4^+^Foxp3^+^ Tregs [47–49] or other types of Tregs [43], as well as a direct effect on CD4^+^ T cells and are in part mediated by IL-10 [48]. In our model, B cells not only inhibited T cells responses to allogeneic pDCs but also directly inhibited proliferation of CD4^+^ T cells stimulated by anti-CD3 in the absence of pDCs *in vitro*. It is unlikely that IL-10 was involved since IL-10 expression by CD45RA^+^ B cells was low and comparable to CD45RA^+^ B cells from non-treated control rats and IL-10 levels in the supernatant of MLRs in the presence or absence of CD45RA^+^ B cells (from Fig. 4D) were not different (data not shown).

CD40 signaling has been shown to be important for Breg development [32,33]. It is therefore possible that in our original model of allograft tolerance induced by blockade of CD40 signaling through treatment with CD40Ig, the emergence of CD8^+^CD45RC^low^ Tregs producing FGL2 (and not of CD4^+^CD25^+^Foxp3^+^ T cells) was promoted [5] but not of Bregs [1]. In CD40Ig-treated recipients, FGL2 possibly promoted allograft tolerance through inhibition of CD4^+^ T cells. In recipients treated with AAV-FGL2 or transferred with CD40Ig-induced CD8^+^Tregs producing high levels of FGL2, where CD40 signaling is intact, the immunoregulatory effects of FGL2 were likely to be a combination of both inhibiting CD4^+^ T cells and promoting Bregs.

In the CD40Ig tolerance-induced model, CD8^+^ Tregs were a source of FGL2 but since CD4^+^ Tregs also produce FGL2 [8], it is possible that Bregs may be induced in other models of transplantation tolerance through FGL2. The induction of Bregs by FGL2 is a new tolerogenic mechanism that underlines the emergence of Bregs as a tolerogenic cell population. FGL2 has the potential to be used in the clinic either as a marker or as an inducer of tolerance.

## Supporting Information

S1 Fig Development of an anti-rat FGL2 antibody and validation of FGL2-recombinant plasmid and AAV vectors. **(A)** Schematic vector construct and nucleic sequence used for recombinant fgl2 plasmid and AAV generation. Complete rat fgl2 sequence was placed after a RSV promoter, an Igk leader, a stalk region and a flag sequence. **(B)** Two rat FGL2 (rFGL2) peptide sequences were used to immunize rabbits and to affinity purify anti-rFGL2 antibodies from rabbit serum. Western blot analysis of denatured human recombinant FGL2-GST protein using rabbit anti-rFGL2 and as a control rabbit IgG from a non-immunized animal. **(C)** HEK293T cells were transfected with FGL2-recombinant or with GFP-recombinant plasmid (pFGL2 and pGFP respectively). FGL2 protein was detected by western blot in the cell lysate and supernatant of transfected cells with the rabbit anti-rFGL2 Ab (n = 3). **(D)** Cytofluorimetry analysis of rat FGL2 protein in transfected HEK293 cells. The left contour plot and black line on histogram show intracellular staining of FGL2+ cells using the rabbit anti-rFGL2 antibody. The right contour plot and filled grey on histogram show signals obtained with control non-immunized rabbit IgG. Data are representative of 3 independent experiments. **(E)** HEK293T cells were transduced or not with AAV-FGL2- at MOI 100 and 10000, and analyzed for FGL2 mRNA expression by quantitative RT-PCR; the spleen was used as a positive control (duplicates, n = 2), and **(F)** for FGL2 protein expression by FACS (black line: anti hFGL2 antibody clone M02; filled grey: isotype control; n = 2). **(G)** Liver (L), spleen (S) and graft (G) samples were harvested 30, 36, and 75 days after AAVFGL2 or AAVGFP injection and analyzed for FLAG-FGL2 expression (172 bp) by nested PCR and Caliper system. Dilutions of FLAG-FGL2 recombinant plasmid were used as positive control.
(TIFF)

S2 Fig Gating strategies for CD4^+^ T cell proliferation in MLRs. **(A)** CD4^+^T were sorted by FACS Aria by gating on TCR and CD4 positive and CD25 negative expression. CD8^+^Tregs were sorted according to CD8^+^ CD45RC^low^ marker expression. pDC were sorted by gating on TCR negative cells, and CD4 and CD45R high expression. All cells were sorted by gating on DAPI negative live cells. Purity was greater than 99%. **(B)** Gating strategy to evaluate CSFE-based CD4^+^CD25^−^ T cell proliferation in an MLR in the presence of allogeneic pDCs based first on morphology (SSC-FSC), exclusion of DAPI positive dead cells, identification of TCR^+^ CD4^+^ T cells and analysis of CFSE.
(TIFF)

S3 Fig Phenotypic characterization of splenocytes, and CD45RA^+^, TCR^+^, pDC cells sorting by FACS Aria. **(A)** Splenocytes were harvested from AAV-FGL2-treated rats with long-term surviving grafts (≥120 days, n = 2), from rats that received a 1st adoptive transfer (1st-transferred, n = 4), and iterative adoptive transfers (2nd transferred, n = 3; 3rd transferred, n = 3; and 4th transferred, n = 2) and from naive animals (n = 11). Splenocytes were counted and analyzed using the indicated markers. Results are expressed in absolute numbers of CD4^+^ T, CD8^+^ T, CD8^+^CD45RC^low^ T, CD8^+^CD45RC^high^ T, B CD45RA^+^ cells and pDCs. Two-Way ANOVA with Bonferroni post-tests p value * <0.05 FGL2-treated recipients vs. naive animals. **(B)** CD4^+^CD25^+^Foxp3^+^T cells were labeled in spleen and graft of splenocytes-transferred (n = 3) vs naive rats (n = 2). **(C)** T cells and pDC were sorted by FACS Aria according to TCR expression and 85C7 Ab-binding respectively, and B cells were sorted by gating on TCR negative and CD45RA positive expression markers, among DAPI negative live cells. **(D)** Purity was greater than 99%.
(TIFF)

S4 Fig FcgammaRIIB expression on B cells and pDCs. **(A)** B cells were sorted by FACS Aria from naive rats (dotted line) or long-term splenocyte-transferred recipients (solid line), stimulated (black line) or not (grey line) with anti-CD40 antibody and CpG ODN for 12h, and labeled for FcgammaRIIB expression or with isotopic control antibody (filled grey). **(B)** pDCs were sorted from naive rats and labeled with FcgammaRIIB antibody or isotopic control antibody.
(TIFF)
