# Supplementary figures and images for: Fibrinogen-Like Protein 2/Fibroleukin Induces Long-Term Allograft Survival in a Rat Model through Regulatory B Cells
Source: PLoS One. 2015 Mar 12;10(3):e0119686. doi: 10.1371/journal.pone.0119686 (PMC4357433; doi:10.1371/journal.pone.0119686)

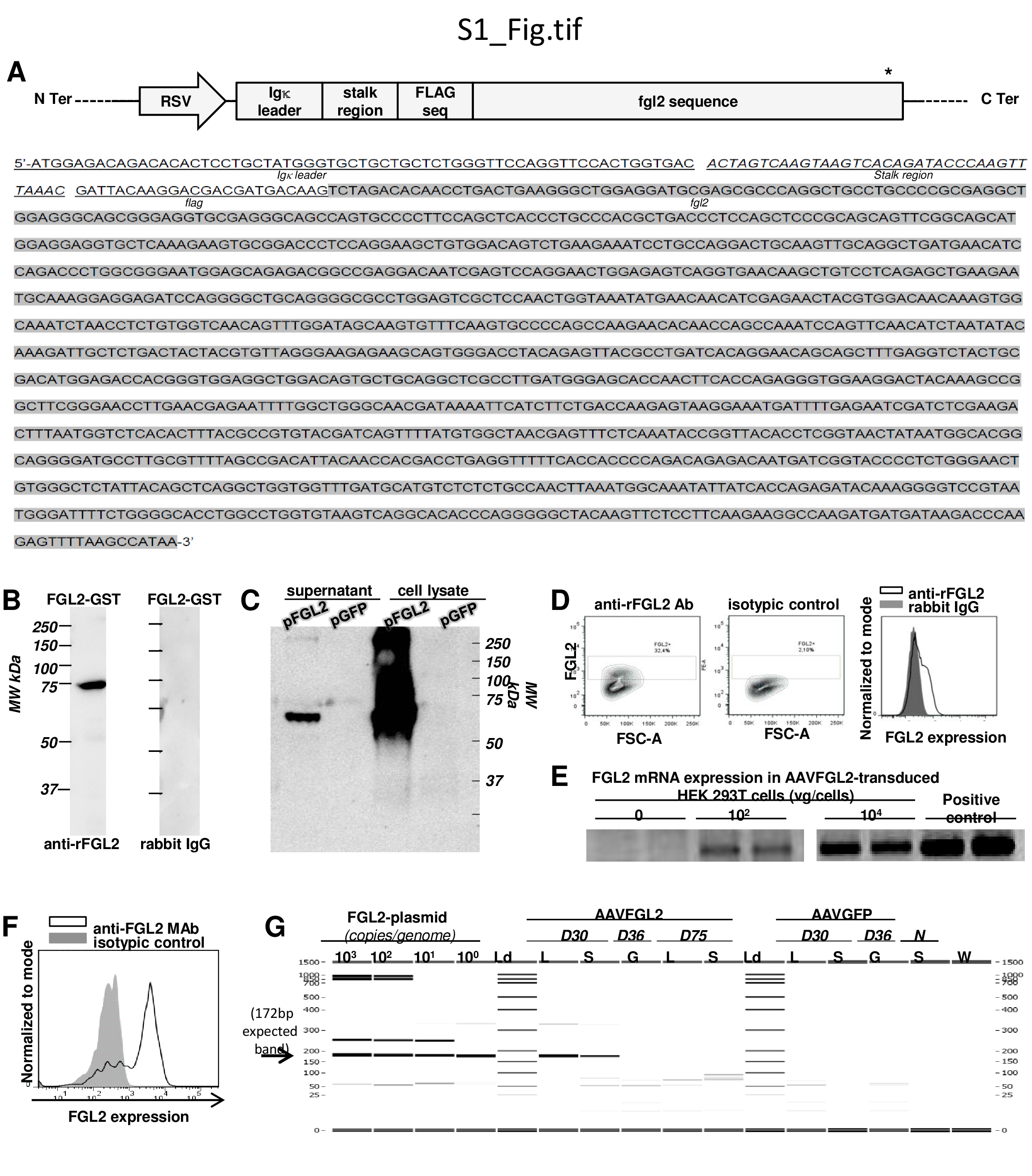

Supplement: S1 Fig — (A) Schematic vector construct and nucleic sequence used for recombinant fgl2 plasmid and AAV generation. Complete rat fgl2 sequence was placed after a RSV promoter, an Igk leader, a stalk region and a flag sequence. (B) Two rat FGL2 (rFGL2) peptide sequences were used to immunize rabbits and to affinity purify anti-rFGL2 antibodies from rabbit serum. Western blot analysis of denatured human recombinant FGL2-GST protein using rabbit anti-rFGL2 and as a control rabbit IgG from a non-immunized animal. (C) HEK293T cells were transfected with FGL2-recombinant or with GFP-recombinant plasmid (pFGL2 and pGFP respectively). FGL2 protein was detected by western blot in the cell lysate and supernatant of transfected cells with the rabbit anti-rFGL2 Ab (n = 3). (D) Cytofluorimetry analysis of rat FGL2 protein in transfected HEK293 cells. The left contour plot and black line on histogram show intracellular staining of FGL2+ cells using the rabbit anti-rFGL2 antibody. The right contour plot and filled grey on histogram show signals obtained with control non-immunized rabbit IgG. Data are representative of 3 independent experiments. (E) HEK293T cells were transduced or not with AAV-FGL2- at MOI 100 and 10000, and analyzed for FGL2 mRNA expression by quantitative RT-PCR; the spleen was used as a positive control (duplicates, n = 2), and (F) for FGL2 protein expression by FACS (black line: anti hFGL2 antibody clone M02; filled grey: isotype control; n = 2). (G) Liver (L), spleen (S) and graft (G) samples were harvested 30, 36, and 75 days after AAVFGL2 or AAVGFP injection and analyzed for FLAG-FGL2 expression (172 bp) by nested PCR and Caliper system. Dilutions of FLAG-FGL2 recombinant plasmid were used as positive control. (TIFF) [file pone.0119686.s001.tiff]

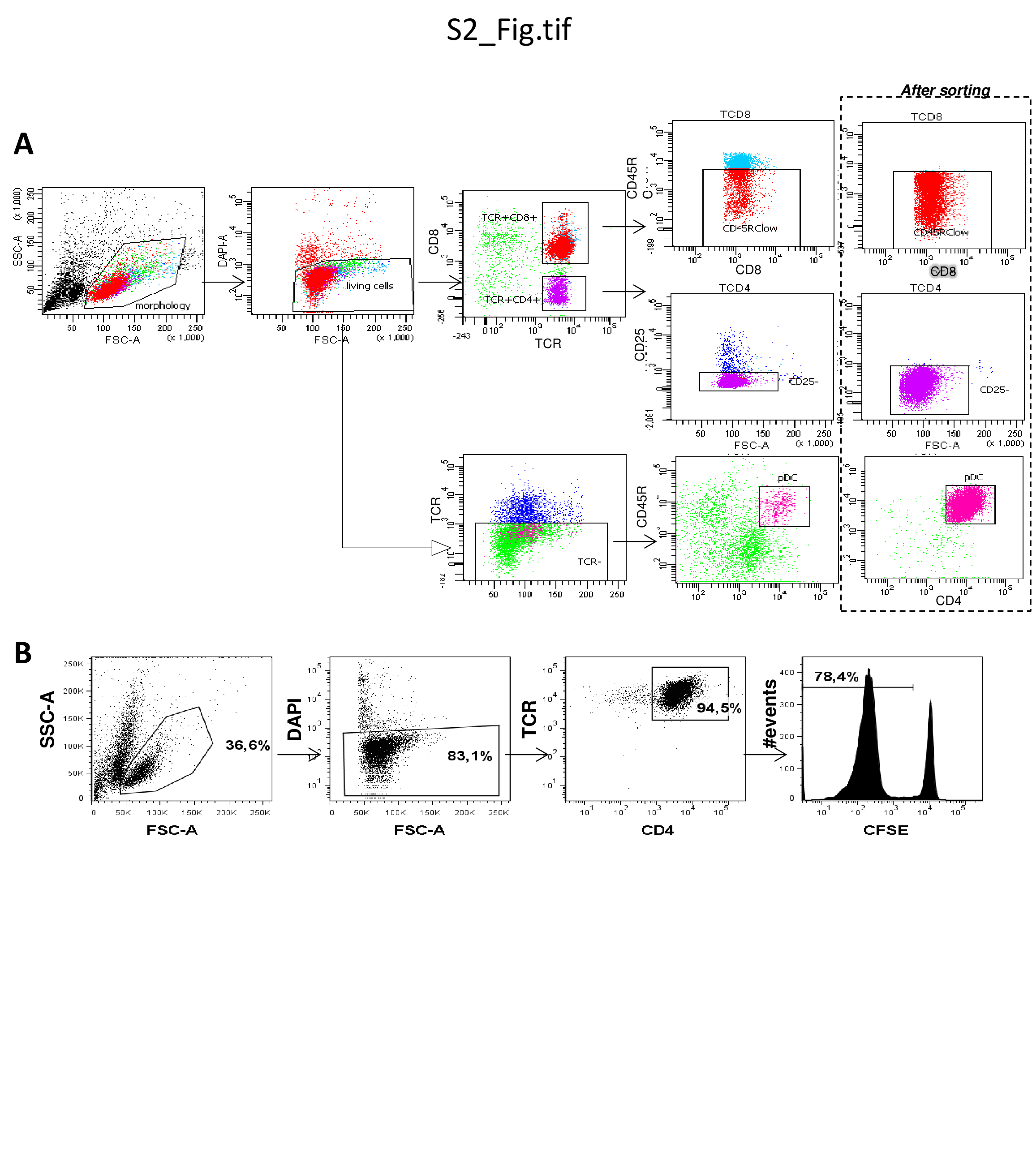

Supplement: S2 Fig — (A) CD4+T were sorted by FACS Aria by gating on TCR and CD4 positive and CD25 negative expression. CD8+Tregs were sorted according to CD8+ CD45RClow marker expression. pDC were sorted by gating on TCR negative cells, and CD4 and CD45R high expression. All cells were sorted by gating on DAPI negative live cells. Purity was greater than 99%. (B) Gating strategy to evaluate CSFE-based CD4+CD25− T cell proliferation in an MLR in the presence of allogeneic pDCs based first on morphology (SSC-FSC), exclusion of DAPI positive dead cells, identification of TCR+ CD4+ T cells and analysis of CFSE. (TIFF) [file pone.0119686.s002.tiff]

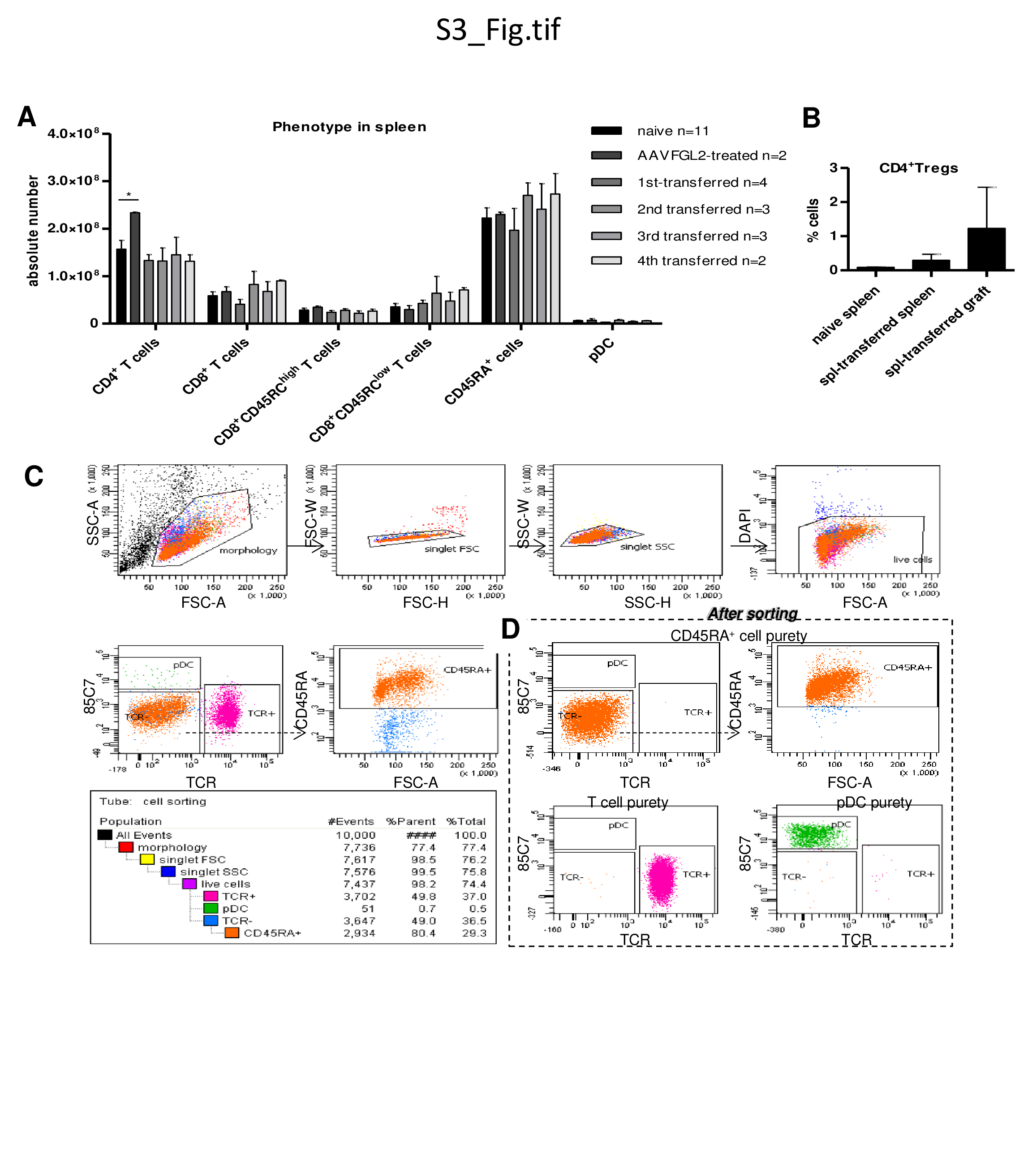

Supplement: S3 Fig — (A) Splenocytes were harvested from AAV-FGL2-treated rats with long-term surviving grafts (≥120 days, n = 2), from rats that received a 1st adoptive transfer (1st-transferred, n = 4), and iterative adoptive transfers (2nd transferred, n = 3; 3rd transferred, n = 3; and 4th transferred, n = 2) and from naive animals (n = 11). Splenocytes were counted and analyzed using the indicated markers. Results are expressed in absolute numbers of CD4+ T, CD8+ T, CD8+CD45RClow T, CD8+CD45RChigh T, B CD45RA+ cells and pDCs. Two-Way ANOVA with Bonferroni post-tests p value * <0.05 FGL2-treated recipients vs. naive animals. (B) CD4+CD25+Foxp3+T cells were labeled in spleen and graft of splenocytes-transferred (n = 3) vs naive rats (n = 2). (C) T cells and pDC were sorted by FACS Aria according to TCR expression and 85C7 Ab-binding respectively, and B cells were sorted by gating on TCR negative and CD45RA positive expression markers, among DAPI negative live cells. (D) Purity was greater than 99%. (TIFF) [file pone.0119686.s003.tiff]

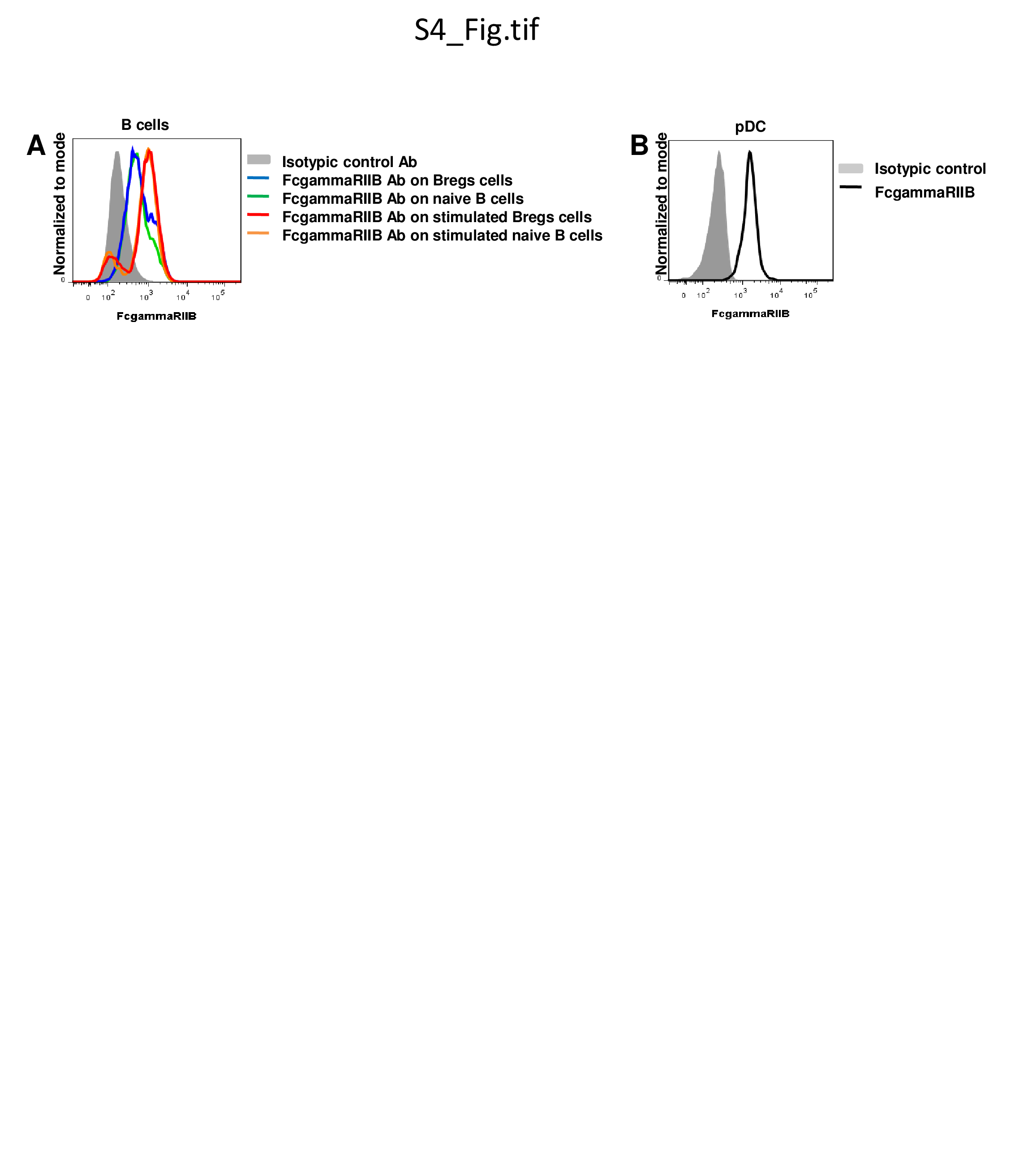

Supplement: S4 Fig — (A) B cells were sorted by FACS Aria from naive rats (dotted line) or long-term splenocyte-transferred recipients (solid line), stimulated (black line) or not (grey line) with anti-CD40 antibody and CpG ODN for 12h, and labeled for FcgammaRIIB expression or with isotopic control antibody (filled grey). (B) pDCs were sorted from naive rats and labeled with FcgammaRIIB antibody or isotopic control antibody. (TIFF) [file pone.0119686.s004.tiff]
